# A multistage controlled intervention to increase stair climbing at work: effectiveness and process evaluation

**DOI:** 10.1186/s12966-016-0371-0

**Published:** 2016-04-11

**Authors:** Alice Bellicha, Aurélie Kieusseian, Anne-Marie Fontvieille, Antonio Tataranni, Nane Copin, Hélène Charreire, Jean-Michel Oppert

**Affiliations:** Institute of Cardiometabolism and Nutrition (ICAN), University Pierre et Marie Curie-Paris 6, Paris, France; Sanofi, 54 rue La Boetie, Paris, 75008 France; Department of Geography, Lab-Urba, Urbanism Institute of Paris, University Paris-Est, Creteil, France; Department of Nutrition, Pitie-Salpetriere University Hospital (AP-HP) University Pierre et Marie Curie-Paris 6, Center for Research on Human Nutrition Ile-de-France (CRNH IdF), Paris, France

## Abstract

**Background:**

Stair climbing helps to accumulate short bouts of physical activity throughout the day as a strategy for attaining recommended physical activity levels. There exists a need for effective long-term stair-climbing interventions that can be transferred to various worksite settings. The aims of this study were: 1) to evaluate short- and long-term effectiveness of a worksite stair-climbing intervention using an objective measurement of stair climbing and a controlled design; and 2) to perform a process evaluation of the intervention.

**Methods:**

We performed a controlled before-and-after study. The study was conducted in two corporate buildings of the same company located in Paris (France), between September, 2013 and September, 2014. The status of either “intervention site” or “control site” was assigned by the investigators. Participants were on-site employees (intervention site: *n* = 783; control site: *n* = 545 at baseline). Two one-month intervention phases using signs (intervention phase 1) and enhancement of stairwell aesthetics (intervention phase 2) were performed. The main outcome was the change in stair climbing, measured with automatic counters and expressed in absolute counts/day/100 employees and percent change compared to baseline. Qualitative outcomes were used to describe the intervention process.

**Results:**

Stair climbing significantly increased at the intervention site (+18.7 %) but decreased at the control site (-13.3 %) during the second intervention phase (difference between sites: +4.6 counts/day/100 employees, *p* < 0.001). After the intervention and over the long term, stair climbing returned to baseline levels at the intervention site, but a significant difference between sites was found (intervention site vs. control site: +2.9 counts/day/100 employees, *p* < 0.05). Some important facets of the intervention were implemented as intended but other aspects had to be adapted. The main difficulty reported by the company’s staff members lay in matching the internal communications rules with critical intervention criteria. The program was maintained at the setting level after the end of the study.

**Conclusions:**

This study shows a successful stair-climbing intervention at the worksite. The main barriers to adoption and implementation were related to location and visibility of posters. Process evaluation was useful in identifying these barriers throughout the study, and in finding appropriate solutions.

**Electronic supplementary material:**

The online version of this article (doi:10.1186/s12966-016-0371-0) contains supplementary material, which is available to authorized users.

## Background

Increasing physical activity (PA) at the population level is a global public health priority [1]. Physical inactivity is recognized by the World Health Organization (WHO) as the 4th leading risk factor for mortality [2]. Also according to the WHO, 43 and 35 % of American and European adults, respectively, were physically inactive in 2011 [3, 4]. The worksite is an important setting for implementing PA promotion programs [5]. Stair-climbing interventions represent a frequent component of such programs. Stair climbing is freely accessible to most population groups, and opportunities to climb stairs are usually available at worksites. Therefore, stair climbing could be easily integrated into daily routine and might contribute to accumulation of PA throughout the day [6].

A common strategy for increasing stair climbing is the use of motivational point-of-decision (POD) prompts. These prompts usually take the form of posters located at a “point-of-choice” (the place at which individuals choose between stairs and the elevator) informing them about the health benefits of stair climbing [7–9]. According to our recent systematic literature review, use of simple motivational prompts increased stair climbing at worksites in only 2 out of 5 studies [10]. However, combining motivational prompts with other types of interventions appeared to be more effective. When prompts were combined with directional signs, stair climbing increased in 5 out of 6 studies [11–15]. Directional signs usually took the form of arrows pointing to the stairs, or footprints informing individuals about a nearby opportunity to use the stairs. One study combined motivational prompts with enhancement in the aesthetics of the stairwell (painting, replacement of doors) and reported an increase in stair climbing [16]. Another effective strategy was to perform two intervention phases. Stair climbing increased during the second phase compared to baseline and to the first phase in 2 out of 3 studies [7, 14, 17]. The second phase of these studies involved motivational signs alone or combined with directional signs. However, little is known about the long-term effects of these interventions, which were evaluated at least 6 months after the end of the intervention in only 3 published worksite studies [18–20]. In addition, very few studies were based on a controlled design [10, 21].

In the field of PA promotion, there is increasing interest in the evaluation of external validity, i.e. the ability of a program to be successfully disseminated and maintained under real-life conditions [22]. Evaluation of external validity involves considering both the effectiveness and the intervention process [23, 24]. Elements of process evaluation are largely under-reported in the field of stair-climbing promotion programs [10]. Only a few studies have reported elements of external validity of stair-climbing interventions, such as the cost of interventions [17, 19], staff expertise [25], consistent implementation of interventions or their adverse consequences [26].

Therefore, the first aim of the present study was to evaluate, under real-life conditions, the short- and long-term effectiveness of a stair-climbing promotion program that included two phases of intervention at a worksite. The second aim was to perform process evaluation of the intervention, describing how the intervention was implemented and adapted to the local setting and the main barriers to its implementation.

## Methods

### Study design and setting

A controlled before-and-after study was conducted in two buildings of the same multinational company (located in the Paris region, France) between September 2013 and October 2014. In both buildings, a health promotion program targeting prevention of non-communicable diseases had been running since early 2013. Health promotion programs at the two sites were developed and supervised by the same team and targeted the same fields of intervention (physical activity, nutrition, sleep habits, stress management, smoking cessation and vaccination). Implementation of the programs was managed locally by dedicated teams and was adapted to each setting (e.g. staff involved or conference topics may have differed between sites). Regarding physical activity, the two programs provided fitness room facilities, fitness classes and open lectures. No stair-climbing intervention had been performed in the past at any site. The stair-climbing study was performed as part of this global program from September 2013 onward. Stair-climbing interventions took place in one building (intervention site) whereas no such interventions were conducted in a second building (control site).

Sampling of the two sites was performed by company management based on their geographic location. They were located 10 km apart in the same urban region, with two distinct buildings at separate locations; however, they were close enough to facilitate study implementation*.* Employees rarely moved from one site to the other, which limited the risk of “contamination”, i.e. of employees from the control site being exposed to stair-climbing interventions. The two sites were similar in terms of working population, nature of the work performed (desk-bound duties) and building design (Table 1). The intervention and control buildings had 6 and 4 floors, respectively. A previous study had shown that the number of floors climbed influenced the rate of stair use with willingness to climb stairs decreasing above two flights [27]. It was thus important to conduct the study in two multi-storey buildings with more than two flights of stairs. The status of intervention or control site was not randomly assigned as one local project team appeared more experienced at conducting a stair climbing intervention, although teams at both sites had similar experience in conducting the other parts of the overall health promotion program.

**Table 1:** Setting description

	Intervention site	Control site
Building occupancy		
Number of employees per month over study period (mean ± SE)	812 ± 5	597 ± 5*
Socio-demographic characteristics		
Mean age / % <45 y	43.4 / 50 %	43.5 / 46 %
Gender (% women)	58 %	59 %
Occupational category (% managers)	75 %	68 %*
Building design		
Number of floors	6	4
Fitness room	Yes	Yes
Fitness classes	Yes	Yes
Company restaurant	Yes	Yes
Baseline level of stair climbing		
Counts/day (mean ± SE)	82.8 ± 2.7	107.9 ± 3.6*

*SE* standard error. Managers = executive, autonomous and integrated officer

*Significantly different from intervention site (*p* < 0.05)

The study was supervised at both sites by a steering committee composed of company executives and researchers. Day-to-day organization of the study was handled at the intervention site by a project team composed of 8 members: 6 were company employees (from 6 different departments: Health, Security and Environment, Occupational Medicine, Communication, Facility Management, Human Resources and Research and Development) and 2 were researchers. Researchers provided guidance on how to conduct the intervention (e.g. number and duration of intervention phases, intervention strategies). The steering committee validated intervention strategies and the project team at the intervention site designed and implemented the intervention. The project team at the control site was informed that a stair-climbing intervention was being performed at the other site, and agreed not to conduct such an intervention during the course of the study.

### Intervention

**Fig. 1:**
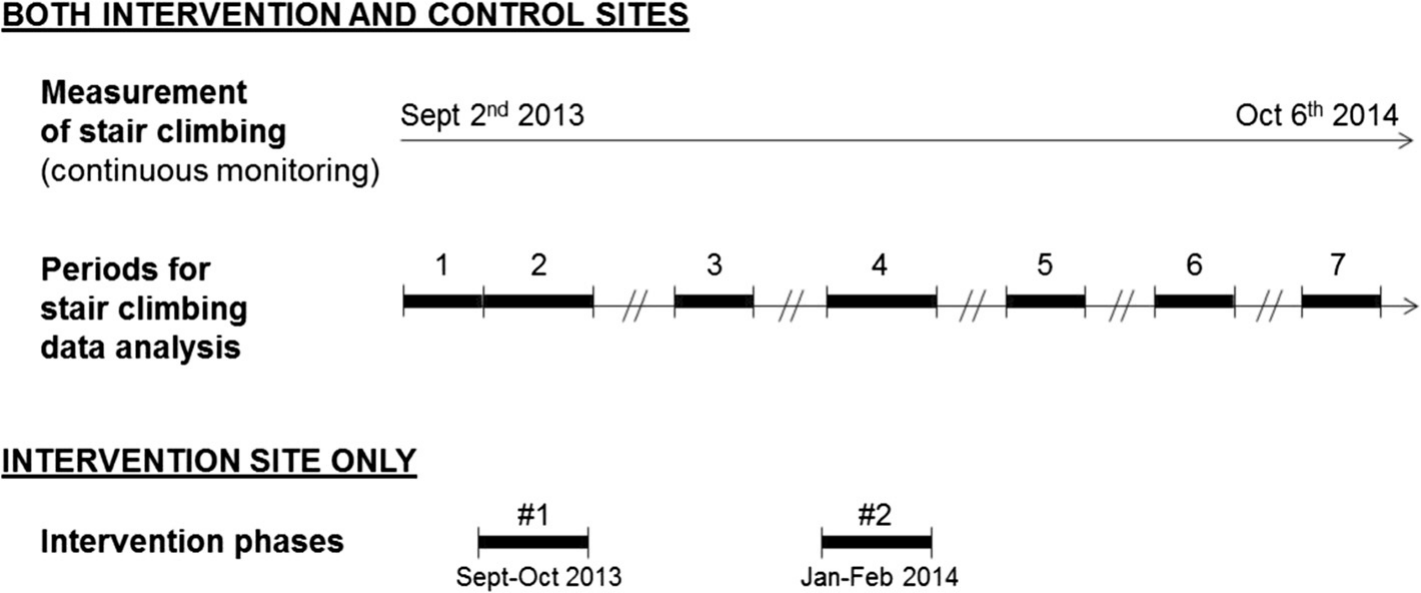
Study design. This study is a controlled before-and-after study which follows an interrupted time-series design. Stair climbing was measured continuously. Numbers represent the 7 study periods: 1 = Baseline period (3-week period); 2 and 4 = Intervention periods (4-week periods); 3, 5, 6 and 7 = Follow-up periods (3-week periods). All study periods corresponded to the same dates at both sites

The definition of the stair-climbing intervention was based on results of our literature review [10]. One main aspect of the intervention was to perform two intervention phases and to combine different strategies to promote stair climbing. The 2-stage design of the intervention was planned from the start of the study. The two intervention phases lasted 1 month each. The first intervention phase combined motivational and directional signs, shown to be an effective combination at worksites [10]. Motivational signs consisted of two large posters (A1 format) located between the stairs and the elevators. They contained a message and a picture representing an anonymous employee or group of employees climbing the stairs. The messages displayed on the posters were written and translated into French by the company and were adapted from previous studies (e.g. “Climbing stairs consumes as many calories per minute as tennis!”) [28]. Four different posters, with different pictures and messages, were created (1 poster per week of intervention). Directional signs consisted of stickers on the walls representing arrows pointing to the stairs. The second intervention phase began 3 months after the first (Fig. 1) and was designed to improve the aesthetics of the stairwells. It included, in addition to the same motivational and directional signs, colorful stickers pasted on to the stair-risers (the vertical part of each step) on every floor. Stickers were colored in blue, orange, green, and beige, alternately. A short message to encourage stair climbing was written in French on the stickers and differed on every floor (intervention materials are available from the corresponding author upon request). The intervention was concurrently conducted for both sets of stairs.

### Measurements

At both sites, two stairs were monitored. At the control site, each stair was close to two elevators and located behind closed doors. At the intervention site, one stair was visible from the elevator and from the lobby, with natural lighting, and was adjacent to only one elevator. The other stair was not visible from the elevator or the lobby; it was located behind closed doors, without natural lighting and distant from four elevators.

Unobtrusive automatic counters that continuously measured stair climbing were located in front of the stairs on the ground floor. Participants were not aware that they were being observed. The counters included a camera using 3D-reconstruction technology: each person moving in the camera’s field of view was reconstructed in three dimensions and registered as a count, with no image stored (3D Counting Ltd, Nantes, France). The counters also distinguished whether a person was entering or exiting the stairwell and whether he/she was going up or down the stairs. Only those who climbed the stairs were registered. Data were stored locally in a processing unit and were downloaded on a regular basis. The correlation between automatic counts and direct observations made on two occasions was Spearman *r* = 0.82 (*p* < 0.001). The target population included all employees working in or visiting the building during the entire study. The study complied with all standards set by the Declaration of Helsinki and did not require formal approval from an ethics committee given the anonymous nature of data as well as the absence of image storage, according to the French Commission on Information Technology and Liberties (CNIL, France).

### Data treatment

At both sites, data from workdays (Monday to Friday, excluding holidays), during opening hours (7:00 a.m. to 9:00 p.m.) and over the same seven time periods were included in the analysis (Fig. 1). The seven time periods included: baseline evaluation, two intervention phases, two short-term follow-up phases (immediately after the end of each intervention phase), a medium-term and a long-term follow-up phase (3 and 7 months after the end of the second intervention phase, respectively).

Stair climbing was expressed as the number of counts per day divided by the number of employees present on site during the same time period (data provided by the Human Resources Department). Changes in stair climbing were described per site (overall analysis of the two stairs) and per stair at the intervention site. Negative effects of intervention were defined as the number of falls occurring on the stairs during the year of the study.

Two minor technical problems occurred, resulting in missing data for one day at the control site during the first intervention phase and 3 days at both sites during follow-up of the second intervention phase. Missing data were replaced by mean values calculated from corresponding weekdays of corresponding months (for example, when data from a Monday in September 2013 were missing, they were replaced by the mean value calculated from the other Mondays in September 2013).

### Statistical analysis

Comparison of characteristics of employees between study buildings was performed using Wilcoxon or Chi-square tests when appropriate. The outcome of interest (stair climbing) was expressed as counts/day/100 employees. A linear mixed model was used to determine the intervention effect (mean change in the intervention site compared to the control site at each time point). Site (intervention, control) and period (1 to 7, corresponding to the baseline period, first intervention phase, short-term follow-up, second intervention phase, short-term follow-up, medium-term follow-up and long-term follow-up) were included in the models as fixed effects. Interaction terms between site and period variables were added (effects at the intervention site compared to the control site for all seven study periods). Results are reported as differences in mean absolute change with 95 % CI and as relative change compared to baseline. Analyses were performed with SAS 9.4 (SAS Institute, Cary, NC).

### Process evaluation

Process evaluation was performed at the intervention site only and the dimensions assessed were adapted from the RE-AIM (Reach, Effectiveness, Adoption, Implementation and Maintenance) framework [23]. In the present study, we focused on adoption, implementation and maintenance dimensions.

Adoption was described as participation of the company’s project team members in defining and implementing the program, and barriers to participation. Both were measured by direct observation (meeting minutes) and through structured interviews. Implementation was the extent to which the interventions were delivered as intended (interventions were defined and conducted by the staff members of the company based on guidelines provided by the research team at the beginning of the study). Data were obtained via regular meetings and structured interviews with staff members. Maintenance was the extent to which stair-climbing interventions were sustained over time at the intervention site. Items used to assess these dimensions are detailed in Additional file 1. We took a narrative approach to describe process evaluation.

## Results

### Effectiveness of interventions

**Fig. 2:**
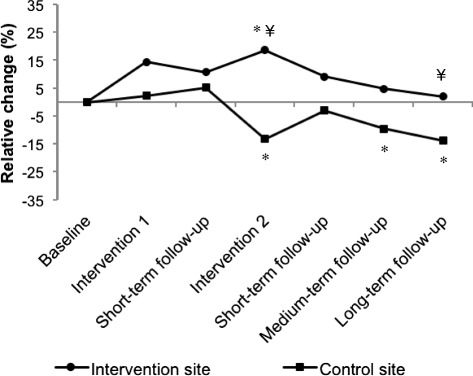
Change in stair climbing in the intervention site and in the control site. * = significantly different from baseline (*p* < 0.05). ¥ = change significantly different from control site (*p* < 0.05)

A total of 36,468 counts were analyzed. Absolute and relative changes in stair climbing at the intervention site and at the control site (two stairs combined) are presented in Table 2 and Fig. 2, respectively. At baseline, stair climbing was almost twice as high at the control site as at the intervention site (Table 1). No significant change in stair climbing was found during the first intervention phase or during short-term follow-up. During the second intervention phase compared to baseline, stair climbing significantly increased by +18.7 % at the intervention site and decreased by -13.3 % at the control site (Fig. 2). The difference between sites was significant (+4.6 counts/day/100 employees at the intervention site compared to the control site, *p* < 0.001) (Table 2). Stair climbing returned to baseline level during short, medium and long-term follow-ups at the intervention site, but decreased significantly at the control site (-9.6 and -13.8 %, during medium and long-term follow-up, respectively). The difference between sites during long-term follow-up was significant (the intervention effect was +2.9 counts/day/100 employees, *p* = 0.019).

**Table 2:** Stair climbing (count/day/100 employees) at the intervention site compared to the control site for each study period

Period	Number of days analyzed	Intervention site		Control site		Intervention effect^a^	p*	p**
		Mean ± SE	Δ (95 % CI)	Mean ± SE	Δ (95 % CI)			
Baseline	15							*<0.001*
Intervention 1	19	12.1 ± 0.6	1.5 (-0.1;3.2)	20.2 ± 0.6	0.4 (-1.2;2.0)	1.1 (-1.2;3.4)	*0.359*	
Short-term follow-up 1	15	11.7 ± 0.6	1.1 (-0.6;2.9)	20.9 ± 0.6	1.1 (-0.7;2.8)	0.1 (-2.4;2.5)	*0.943*	
Intervention 2	20	12.6 ± 0.5	2.0 (0.4;3.6)	17.2 ± 0.5	-2.6 (-4.3;-1.0)	4.6 (2.3;6.9)	*<0.001*	
Short-term follow-up	15	11.5 ± 0.6	1.0 (-0.8;2.7)	19.2 ± 0.6	-0.6 (-2.3;1.1)	1.6 (-0.9;4.0)	*0.211*	
Medium-term follow-up	15	11.1 ± 0.6	0.5 (-1.2;2.2)	17.9 ± 0.6	-1.9 (-3.6;-0.2)	2.4 (0;4.9)	*0.055*	
Long-term follow-up	15	10.8 ± 0.6	0.2 (-1.5;1.9)	17.1 ± 0.6	-2.7 (-4.5,-1.0)	2.9 (0.5;5.4)	*0.019*	

All estimates were from the linear mixed regression model, using site and study period as fixed effects

*95* % *CI* confidence interval at 95 %, *SE* standard error

Δ estimated mean change between baseline and the study period

^a^ estimated effects in intervention site compared with the control site

*p-value from the interaction effects (i.e. test of difference in change for intervention versus control at each period)

**p-value from the overall interaction effect (i.e. test of difference in change for intervention versus control over time)

**Fig 3:**
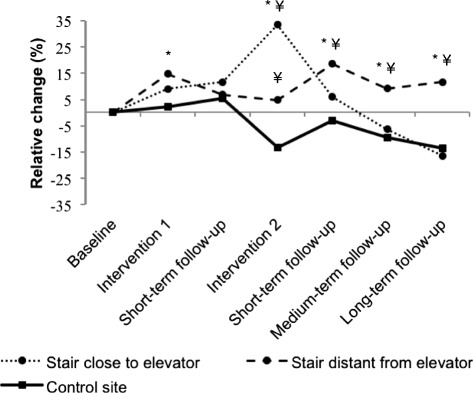
Change in stair climbing in two stairs of the intervention site and in the control site. * = significantly different from baseline (*p* < 0.05). ¥ = change significantly different from control site (*p* < 0.05)

Figure 3 shows changes in stair climbing for each stair at the intervention site (one stair close to the elevator, the other distant from the elevator). Compared to the control site, stair climbing increased during the second intervention phase for both stairs (+29.8 and +6.6 % in the stairs close to and distant from the elevator, respectively). However, stair climbing remained high compared to baseline and compared to the control site during follow-up only in the stair that was distant from the elevator (+7.4 % during long-term follow-up).

One fall in the stairs (without major injury) was reported during the year before the study and one during the year of the study, suggesting that the increased use of stairs did not cause any additional accident.

### Process evaluation of the intervention

#### Adoption dimension

Four company employees participated in the design and implementation of the stair-climbing interventions (program director, occupational physician, employees from the communications and facility management departments). Implementation of the first intervention phase was described as “difficult” by two employees and “rather difficult” by two others. The main barrier concerning the location of posters was related to internal rules of communication. The point-of-choice, suggested by the research team for its high visibility was dedicated to corporate communications and was therefore considered inappropriate for promoting stair climbing. The implementation of the second intervention phase was described as “rather easy” or “rather difficult” by 3 and 1 employees, respectively. They found that installation and removal of stickers required time and workforce investment.

#### Implementation dimension

The framework provided by the research team was followed since two intervention phases using the recommended strategies were conducted. The recommended strategy for the first phase was to introduce posters located at the point-of-choice and visible directional signs. Adaptations mainly involved visibility of the signs: posters were located in a less visible section of the building and directional signs were not easily seen given their full compliance with the corporation graphics charter. The recommendation for the second intervention phase was to improve the aesthetics of the stairwell by using music or artwork. The introduction of artwork and music in the stairwell was considered too expensive and complex to organize. Therefore, colorful stickers enhancing the aesthetics of the stairs were used.

#### Maintenance dimension

Stair-climbing interventions were maintained after the end of the study. Two additional intervention phases (in October 2014 and April 2015), designed by the company in collaboration with a communications agency, were carried out (using new posters with different messages and pictures). Moreover, stair-climbing interventions were included as one of the main health promotion interventions deployed at other sites by the company.

## Discussion

We evaluated the effectiveness and described the process of a stair-climbing promotion program that included two phases of intervention in a worksite setting. Stair climbing significantly increased at the intervention site by 19 % during the second intervention phase, while it decreased at the control site.

This range of increase in stair climbing is in line with previous studies conducted at worksites, which reported a median change in stair climbing of +17 % [10]. From our data, we estimated that around 35 more persons climbed the stairs each day during the second intervention phase. Indeed, our results strengthen the interest of performing a two-stage intervention within a worksite setting. In this setting, around 35 % of previous studies reported no effect of the first intervention phase, which consisted most often of motivational prompts [10]. Point-of-decision prompts function by interrupting the habitual behavior of choosing the elevator [7]. Thus, a single intervention phase could help engaging in new activities, but repeated interventions may be necessary to create new sustainable habits. Our second intervention phase used the same posters and directional signs as that of the first phase; in addition, colorful stickers were applied to the stair risers. Therefore, we could not identify whether it was the repetition or the novelty of the intervention that positively influenced stair climbing. Few previous studies consisted of 2 intervention phases, and most of them measured stair use (i.e. a combination of climbing up and going down stairs) but not stair climbing in particular [7, 14, 17, 25, 29–32]. Among studies that found no change in stair use during the first phase, three used a different strategy during the second phase and reported a positive effect [7, 17, 29]. The only study that used the same strategy reported no change in stair use during the second phase [25]. Therefore, changing strategy for the second intervention phase appears to be particularly important if the first intervention fails to substantially increase stair climbing.

Our second intervention phase aimed to modify the aesthetics of the stairwell. This strengthens the findings of some previous studies that found promising results using this strategy [16, 20, 29, 33]. Three of those studies performed stairwell enhancement in addition to POD prompts. The only study that began the interventions with stairwell enhancement found no effect until prompts were added [20]. Colorful stickers applied to stair-risers on every floor of the building, as used here, appear easier to implement than previous initiatives introducing artwork and music in the stairs, replacing wooden doors by glass doors, or repainting the walls with new colors [16, 20, 29, 33]. The strong visibility of the stickers was likely important in explaining their effectiveness. In contrast, our first intervention phase may have lacked visibility and may have been difficult to distinguish from corporate communications. This suggests that breaking a routine at work requires highly visible interventions capturing employee attention.

Another important feature of our study was that we compared the effectiveness of the intervention for two stairs with differing design features. We found that the second intervention phase was effective for both stairs of the intervention site. This suggests that stair climbing can be increased whether the stairs are visible or not from the elevator, and that interventions should not be performed only in the most visible stairs. Interestingly, the increase in stair climbing was almost 5 times higher in the stair visible from the elevator that had natural lighting and was adjacent to only one elevator. Over the long term, however, the effect persisted only in the other stair (not visible from the elevator, located behind closed doors, without natural lighting and distant from four elevators), even though the effect size was limited. Improving the aesthetics of the stairs was thus less effective during the intervention period in the less attractive stair but more effective over the long-term. A possible explanation might be that employees positively changed their awareness of and attitude toward the distant stairs. Although less attractive, this stair was most convenient for joining the office spaces and meeting rooms. Employees might have become more willing to take this stair even after the intervention was over.

An important aim of our study was to describe the intervention process. A major barrier to adoption by employees was identified, i.e. trying to locate the posters in a location usually dedicated to corporate communications. However, this problem was solved later in the study by discussion between members of the steering committee and those of the project team. This example is in accordance with national guidelines recognizing management support as being a key factor in the success of health promotion programs [34]. It also suggests that, in the current challenging economic context, companies may be reluctant to communicate on these programs that could be considered by some as inappropriate.

Regarding implementation, the company adhered to overall aspects of the initial program: they conducted two intervention phases and used recommended strategies. However, some critical aspects of the intervention were adapted by the company. The main adaptations were the lack of visibility of motivational and directional signs and the use of colorful stickers instead of artwork and music to improve the aesthetics of the stairs. This raises the problem of feasibility of major stairwell modifications at worksites and highlights the importance of finding easy-to-implement interventions. Pasting colorful stickers on the stair-risers is an interesting option.

The program was maintained at the setting level, and will likely by deployed in other buildings of the same company in the near future, which is a very positive finding. This highlights the need for ongoing dialogue between company staff and researchers during implementation to overcome challenges associated with such interventions.

Strengths of our study included the controlled design in two similar buildings, objective monitoring of stair climbing over extended periods, length of follow-up and description of the adoption, implementation and maintenance of the intervention. Some limitations should be mentioned. Results were expressed as absolute counts of stair climbing and were difficult to compare with previous studies, almost all of which had expressed stair use as a percentage compared to elevator use [10]. One reason why we did not measure elevator use was that both buildings under study had a basement and the elevators not only went up from the ground floor to higher floors but also went down to the basement. Although we could have distinguished those entering or exiting the elevator, it would have been impossible to differentiate elevators going up and down, and thus it was impossible to calculate stair climbing as previous studies (percent of stair climbing compared to elevator climbing). Another limitation was the inability of automatic counters to identify personnel characteristics such as gender and age, and whether individuals were regular workers in the building or were visitors.

## Conclusions

In summary, our findings strengthen the interest of repeated interventions at worksites, as well as enhancing of stairwells to substantially increase stair climbing. They also suggest that stair-climbing interventions can be conducted for different types of stairs (e.g. close to or distant from elevators), increasing the potential of transferability of these interventions. The main barriers to adoption and implementation were related to location and visibility of posters. Process evaluation was useful for identifying such barriers throughout the study and for finding appropriate solutions. Maintenance of the intervention at the setting level is encouraging. Further studies should be conducted at other types of worksites and investigate further the Reach and Adoption dimensions of transferability.

## Additional file

Additional file 1: Items used for process evaluation of the intervention. (DOC 33 kb)
