# Supplementary material for: A multistage controlled intervention to increase stair climbing at work: effectiveness and process evaluation
Source: Int J Behav Nutr Phys Act. 2016 Apr 11;13:47. doi: 10.1186/s12966-016-0371-0 (PMC4827230; doi:10.1186/s12966-016-0371-0)
Supplement: Additional file 1: — Items used for process evaluation of the intervention. (DOC 33 kb) [file 12966_2016_371_MOESM1_ESM.doc]

**Additional file 1**

| **Dimensions assessed** | **Evaluation question** | **Indicator** | **Measurement tool** | **Questions** |
| --- | --- | --- | --- | --- |
| Adoption | To what extent did the staff members participate in the intervention? | Staff members’ participation,  barriers to adoption | Meeting minutes  + Interviews  with staff members | Have you participated in the definition and/or implementation of stair-climbing interventions? *🞏 Yes 🞏 No*  If so, how would you describe the implementation of those interventions?  *🞏 Easy 🞏 Rather easy 🞏 Rather difficult 🞏 Difficult 🞏 No opinion*  What main barriers have you met? ……………  What did you think of stair-climbing interventions one year ago (in terms of interest for employees)? *🞏 Very good idea 🞏 Relatively good idea 🞏 Relatively bad idea 🞏 Very bad idea 🞏 No opinion*  What do you think of these interventions now? *🞏 Very good idea 🞏 Relatively good idea 🞏 Relatively bad idea 🞏 Very bad idea 🞏 No opinion* |
| Implementation | Were the interventions  delivered as intended? | Consistent implementation  of program,  staff expertise, cost,  time needed for implementation | Meeting minutes  + Interviews  with staff members | The interventions delivered are different from those suggested by the researchers. Why have you made those changes? ……………  Those interventions were performed in addition to your usual duties. How would you describe this extra work?  *🞏 Easy to integrate into my work days 🞏 Relatively easy to integrate*  *🞏 Relatively difficult to integrate 🞏 Difficult to integrate 🞏 No opinion*  About how many hours would you say you worked for those interventions? ............. |
| Maintenance | To what extent were the interventions maintained after the end of the collaboration? | Maintenance  (setting level) | Interview with project director | Have other stair-climbing interventions been implemented since the end of the study? *🞏 Yes 🞏 No*  If so, what interventions? ……………  Do you plan to perform other such interventions in the future? *🞏 Yes 🞏 No* |
